# Novel Kraft Softwood Lignin-Derived Carbon Quantum Dots: Synthesis, Characterization, and In Vitro Cytocompatibility

**DOI:** 10.3390/nano14121029

**Published:** 2024-06-13

**Authors:** Eli Christoph, Lu Yu, Steven D. Newby, Michael A. Rivera Orsini, Jakob Scroggins, David J. Keffer, David P. Harper, Madhu Dhar

**Affiliations:** 1 Material Science and Engineering, University of Tennessee, Knoxville, TN 37996, USA; echris11@vols.utk.edu (E.C.); yu11@ornl.gov (L.Y.); jscrogg4@utk.edu (J.S.); dkeffer@utk.edu (D.J.K.); 2 Tissue Engineering and Regenerative Medicine, Large Animal Clinical Sciences, College of Veterinary Medicine, University of Tennessee, Knoxville, TN 37996, USA; snewby@utk.edu (S.D.N.); mriverao@utk.edu (M.A.R.O.); 3 Center for Renewable Carbon, School for Natural Resources, University of Tennessee, Knoxville, TN 37996, USA; dharper@utk.edu

**Keywords:** lignin, carbon quantum dots (CQDs), human mesenchymal stem cells (hMSCs), proliferation

## Abstract

Carbon quantum dots (CQDs) have been investigated for biomedical applications in medical imaging due to their fluorescent properties, overall long-term stability, and excellent cytocompatibility and biocompatibility. Lignin is an organic polymer in the tissues of woody plants. It is also considered a byproduct of the wood and pulp industries. Hence, it presents as a renewable source of carbon nanoparticles. In this study, we report the synthesis and material and biological characterization of two colloidal suspensions of CQDs in water derived from lignin-based carbon. One was the native form of CQDs derived from lignin carbon, and the second was doped with nitrogen to evaluate material differences. Material characterization was carried out using various commonly used techniques, including Fourier transform infrared spectroscopy (FTIR), emission and absorbance spectra, zeta potential, transmission electron microscopy (TEM), and scanning electron microscopy (SEM). Thin films of CQDs were formed on glass and silicon substrates to assess the in vitro cytocompatibility with human mesenchymal stem cells (hMSCs). Observations suggest that the two forms of CQDs promote cell attachment within 24 h and sustain it for at least 7 days. The overall structure and shape of cells suggest a lack of any adverse or toxic effects of CQDs. The data lay down the novel foundation to support the use of lignin-derived CQDs in tissue engineering applications.

## 1. Introduction

Softwood lignin is an organic copolymer found in the cell walls of softwood plants that contributes to mechanical support, water transport, and stress resistance. Lignin can be extracted through several delignification methods using aqueous or organic solvents [1]. As an abundant, renewable, and naturally occurring aromatic polymer, lignin is a renewable material to use as a carbon feedstock. Lignin accounts for approximately 15–35% of the weight of lignocellulosic biomass and can help produce fuels and chemicals as a significant renewable resource. Lignin is an environmentally friendly, relatively inexpensive, and accessible material. Extracting pure lignin enables the synthesis of high-value biomaterials with biomedical applications. By virtue of its antioxidant and antibacterial properties, lignin is being investigated for various biomedical applications such as bioimaging, 3D printing, and tissue engineering [2,3].

Lignin is heterogeneous, with a complex structure of phenylpropanoid moieties consisting of monolignols. Monolignol units include *p*-coumaryl, sinapyl, and coniferyl alcohols [4,5]. The most prominent linkage in lignin is β-O-4 aryl ether bonds, contributing up to 50% of the linkage in softwood lignin. Additional functional groups within lignin include benzyl alcohol, aliphatic and aromatic hydroxy, methoxy, and carbonyls. The isolation of lignin using the kraft pulping process results in the cleavage of linkages that hold the phenylpropane units together. New bonds can be formed as a result of condensation; however, carbon–carbon bonds tend to remain undisturbed [6]. Previous work demonstrated that the control of the lignin feedstock, pulping conditions, and thermal processing could lead to careful control of the nano-to-microstructure in the resulting carbon materials [7,8,9,10,11].

Nanomaterials can be broadly classified into organic, inorganic, and carbon-based particles in nanometric scale [12]. Nanoparticles exhibit different properties compared to their larger-size counterparts and, hence, have been proposed to have a significant role in biomedicine. Carbon quantum dots, a type of carbon-based nanoparticles, have been investigated for biomedical imaging applications due to their fluorescent properties, long-term stability, and potential cytocompatibility and biocompatibility [13]. The fluorescent properties of the CQDs are attributed to the quantum confinement effect that occurs in quantum dots. This phenomenon is described as the particle radius being smaller than the Bohr excitation radius. As a result, electron–hole pairs develop due to excitation and are forced to recombine since the electrons cannot escape the quantum dot and flow as conduction electrons. When electrons and holes recombine, excess energy is released through visible fluorescence. The emitted photon wavelength and intensity are directly dependent on the particle size of the CQDs. A smaller quantum dot will cause an electron to have more energy to release than a relatively larger quantum dot, thus changing the color towards a more violet, fluorescent emission.

The usage of carbon quantum dots in tagging a desired cell through their fluorescent properties has increased in recent years for applications in tumor treatment and tissue labeling [14,15]. The potential of carbon-based nanoparticles, specifically graphene and CQDs in regenerative medicine and tissue engineering, has been realized following the discovery of these nanoparticles in 2004. The potential to fabricate various derivatives and forms of graphene and CQDs with specific material physicochemical properties has facilitated this area of research. Their enhanced properties, such as high reactivity, strength, surface area, sensitivity, stability, and functionalization, have made them important for tissue engineering and regenerative medicine studies [16]. Shang et al. reported the synthesis of graphene quantum dots from high-purity graphite rods through a facile electrochemical method. Subsequently, they demonstrated the uptake of graphene quantum dots in human neural stem cells via endocytosis [17]. Importantly, cellular uptake did not affect the performance and function of the cells. The ability of the quantum dots to be absorbed by the cells was found to be dependent on the particles’ surface charge. Chung et al. reported that CQDs with high positive surface charge were cytotoxic, whereas nanoparticles with a relatively low magnitude of the positive surface charge were cytocompatible with human mesenchymal stem cells (hMSCs) and were ingested by the cells [18]. Yan et al. investigated the effect of the surface charge of mesoporous silica nanoparticles on hMSCs and 3T3-L1 cells. They found that the nanoparticles were biocompatible and did not affect cell proliferation and differentiation but that the ability of cells to ingest the nanoparticles depended on the type of cell and the magnitude of positively charged particles. A clear correlation was observed between the surface charge and the number of tagged 3T3-L1 cells. Their results suggest that the ability of cells to ingest nanoparticles can be regulated through the modulation of the positive surface charge of nanoparticles [19]. Electropositive citric acid-polyethyleneimine-derived CQDs have been reported to be biocompatible with human MSCs and are ingested by MSCs to act as fluorescent cell markers. This research has significant promise in understanding MSCs’ behavior in vivo through using CQDs as trackers for MSC migration [20]. Extensive studies, including work from our laboratory, have reported the ability of specific carbon-based materials to promote cell proliferation and lineage-specific differentiation [21].

It has also been reported that negative surface-charged nanoparticles can bind to cellular membranes or be taken up by the cells. These processes can be attributed to the zeta potential of nanoparticles. Cellular membranes consist primarily of negatively charged domains with cationic sites. The negatively charged particles can bind to these cationic sites via electrostatic interactions, which can result in cellular adhesion and potential differentiation [22,23,24]. For instance, Mahmoud et al. reported several nanoparticles with a negative zeta potential to induce cell adhesion and osteogenic differentiation of rat MSCs [25].

Carbon quantum dots are typically synthesized by hydrothermal/solvothermal methods using any carbon sources, including foods (honey, sugar beet molasses), chemicals (citric acid, citrate salts, acrylic acid), plants, and graphene [26]. Previous data from our lab report the development of lignin-derived carbon nanoparticles for application in high-performance super capacitors [8,27]. The application of these CQDs to control hMSC proliferation and bioactivity for tissue engineering scaffolding has yet to be investigated. Studies have been performed to show a positive impact of CQDs on in vivo wound healing using citric acid-derived CQDs. A hydrophilic aqueous base cream containing CQDs was applied to the wounds of rats in an animal model study and was shown to promote wound healing by up to a 30% faster healing rate compared to the untreated animals in the control group [28]. Praseetha et al. showed the ability of aloe vera in junction with beetroot extract-derived CQDs with a negative surface charge to have antimicrobial properties and promote wound healing in a zebrafish model in vivo [29]. In a bone tissue engineering study, adult human bone marrow-derived MSCs responded to both the adenosine and aspirin-derived CQDs in vitro. As expected, CQDs were cytocompatible and underwent healthy cell endocytosis. The authors used osteogenic transcription and matrix mineralization assays to show that the CQDs triggered the MSCs to undergo osteogenic differentiation. The precursors alone did not stimulate any cell response, suggesting that the CQDs drove cell proliferation and differentiation observed in this study [30]. In summary, multiple, relatively simple carbon precursors have been investigated as a source of CQDs, which have shown promise in cell and tissue regeneration. The ability to take CQDs to the next step and control the fate of MSCs through nanoscale patterning of a scaffolding surface remains an outstanding challenge in the field of regenerative medicine.

Potential breakthroughs in regenerative medicine’s abilities to reach popular clinical applications depend on cost efficiency, reproducibility, ease of production, and environmental friendliness. Lignin, an abundant and inexpensive natural carbon-based resource from the tissue of woody plants, holds promise to be a long-term source material for CQDs and other biomaterials with regenerative medicine applications. This can be attributed to the renewable and abundant source of lignin being woody plants, the inexpensive cost of raw material, and the ease of CQD synthesis from a lignin precursor.

Efforts have been undertaken to explore the utilization of lignin as a source for producing carbon dots. However, most of them directly use the lignin as precursors [31,32]. Due to lignin’s complex polymer structure, it is challenging to precisely control the synthesis process and the properties of the carbon dots produced directly from lignin. In this study, we report the novel use of lignin-based activated carbon as the precursor for producing two separate suspensions of kraft softwood lignin-derived CQDs in water, and the novelty of assessing their cytocompatibility with hMSCs.

Following synthesis, the CQDs were characterized through Fourier transform infrared spectroscopy (FTIR), emission, absorbance spectra, zeta potential, transmission electron microscopy (TEM), and scanning electron microscopy (SEM). A key difference between the two forms of lignin-derived CQDs was the nitrogen doping of one form relative to the other. This modification was performed to investigate whether the addition of amino groups onto the surface of nanoparticles affected cell attachment. Thin films of lignin-derived CQDs were formed on glass and silicon substrates to assess the cytocompatibility and cell response to CQDs in vitro. This strategy is distinct from the published reports described above. Our experiments are designed to predict the response of hMSCs to the surface features of CQD films rather than the response that is triggered when nanoparticles are taken up by the cells in the culture. Using F-actin staining, we observed that both forms of CQDs promoted cell attachment within 24 h, and the attachment was sustained even after 7 days, confirming the cytocompatibility of CQDs. Data obtained will guide the use of CQDs as a biomimetic component in 3D printed scaffolds, with a potential to be used as a medical device in vivo, confirming that both the forms of lignin-derived CQDs have strong promise to be used in tissue engineering projects [33].

## 2. Materials and Methods

Indulin^®^ AT kraft softwood lignin was purchased from Ingevity Ltd. (North Charleston, SC, USA). Polyethyleneimine was purchased from Polysciences (Warrington, PA, USA), and silicon wafers were obtained from Electron Microscopy Services (Hatfield, PA, USA). All general lab chemicals, biochemicals, and disposables were purchased from Thermo Scientific and Fisher Scientific (Waltham, MA, USA) unless reported otherwise.

### 2.1. Carbon Quantum Dot Synthesis

Lignin-based activated carbons served as the precursor of CQDs. Kraft softwood lignin was pyrolyzed in a tube furnace at 800 °C under an N_2_ environment for one hour to obtain the carbonized char. Then the prepared carbons were subjected to physical steam activation at 800 °C for one hour to generate the lignin-based activated carbons.

Following precursor synthesis, two lignin-derived CQD colloidal suspensions in water were prepared. The two forms are referred to as S1 and S2 throughout this study. The S1 and S2 forms of CQDs were prepared using a bottom-up approach to solvothermal synthesis [26]. To prepare the S1 form, 1.5 g of lignin-based activated carbon was mixed with 6 mL of DMF and 3 mL of H_2_O_2_ in a bomb reactor. The solution was mixed thoroughly and placed in a furnace at an initial temperature of 30 °C for five minutes. The furnace temperature was then raised to 180 °C at a ramp rate of 10 °C per minute and held at 180 °C for 1.5 h. After naturally cooling down, the bomb reactor was rinsed with DI water until a total of 200 mL of aqueous solution was obtained. The solution was then divided into four 50 mL vials and centrifuged four times for ten minutes at 10,000 rpm each. This resulted in the separation of the solution into CQDs dispersed in water at the top and excess carbons with big particle sizes settling at the sides and bottom of the vials. An automatic pipette was used to extract the translucent solution selectively and avoid introducing the big carbon particles. This resulted in 100 mL of CQDs solution. The weight of the CQD solution was then recorded using a microbalance. The solution was placed in a furnace at 103 °C for 48 h to evaporate the aqueous solvent. The mass of the beaker and the remaining carbon material were recorded, providing us with the mass of the CQDs. Then, 100 mL of milli Q water was added and stirred to disperse the CQDs into the aqueous solvent. The resultant weight concentration of the S1 form of CQDs in solution was 1.37 × 10^−4^ g·mL^−1^. The S2 form of CQDs was synthesized using the same process described above, with one modification. The lignin-based activated carbon was submerged in 8M nitric acid (HNO_3_) solution for 6 h at 80 °C before synthesizing the CQDs. S2 dispersion of 1.37 × 10^−4^ g·mL^−1^ in MilliQ water was obtained identical to S1. The fluorescence of the S1 and S2 dispersions was observed under a 365 nm wavelength UV light, confirming the presence of CQDs in the solution, with minimal extra carbon material remaining in the beaker.

### 2.2. Carbon Quantum Dot Characterization

#### 2.2.1. Physical Characterization

A Biotek Synergy H1 microplate reader (Agilent, Santa Clara, CA, USA, https://www.agilent.com/, accessed on 6 June 2024) was used to gather emission spectra of S1 and S2 dispersions using a light with a 350 nm wavelength, which can cause CQDs to fluoresce [34]. In total, 200 µL of each sample was used to evaluate the Emission spectra. The excitation wavelength was 350 nm, with an instrument gain value set at 80 and the step count of the emission peak at 2. Emission spectra were then collected to assess the fluorescent intensity.

A PerkinElmer Lambda 650 UV/VIS Spectrometer (PerkinElmer, Shelton, CT, USA, https://www.perkinelmer.com/, accessed on 6 June 2024) was used to gather the absorption spectra of S1 and S2 forms. A background run of a blank cuvette was first obtained. Subsequently, S1 and S2 dispersions in DI water were analyzed. Absorbance scans were collected, and the percent transmittance of waves over 250 to 800 nm was obtained. 

A PerkinElmer FT-IR spectrometer was used to obtain the FTIR spectra for S1 and S2 dispersions. The experiment was performed as per the manufacturer’s instructions (https://www.perkinelmer.com/). Briefly, 150 µL of each S1 and S2 dispersions were loaded onto a clean piece of aluminum foil. The droplets were dried over a period of 24 h at 80 °C. FTIR was performed three times for each dispersion. A background run of aluminum foil was performed for comparison.

A Malvern Panalytical instrument (Malvern Panalytical, Malvern, UK, https://www.malvernpanalytical.com/en/, accessed on 6 June 2024) was used to collect the average zeta potential of 1 mL of each S1 and S2 dispersion.

S1 and S2 dispersions were characterized using the above methods, and each batch was characterized to ensure that the CQD properties were unchanged. Once the properties were confirmed and no variation in different batches was observed, only then they were used in the applications described below.

#### 2.2.2. Microscopy

Both the S1 and S2 forms of CQD dispersions were further characterized by SEM and TEM to gain an understanding of the particle size and morphology. For imaging, a thin film of nanoparticles on an appropriate substrate was required. Hence, each dispersion was coated on a polyethyleneimine (PEI) coated silicon substrates. PEI is considered a binding agent for carbon nanoparticles and, is cytocompatible and does not affect cell behavior, including proliferation and differentiation potentials [35]. A stock solution of 1 g·L^−1^ PEI in 200 milli-Q water was prepared. The solution was mixed for 15 min to dissolve PEI. The silicon substrates were submerged in the PEI solution for 24 h. The samples were rinsed thoroughly with milli-Q water and dried using compressed air. Then, 150 µL of the S1 and S2 CQD dispersions were added to form a nanoparticle film. Samples were dried for 24 h at 80 °C and imaged using a Zeiss Auriga Crossbeam FIB/SEM (Ziess, Jena, Germany) instrument at the University of Tennessee’s Institute for Advanced Materials and Manufacturing.

For TEM, 20–30 μL of each dispersion was loaded onto a TEM grid. Each sample was stained with approximately 30 µL of UranyLess EM Stain (Electron Microscopy Sciences, Hatfield, PA, USA, https://www.emsdiasum.com/, accessed on 6 June 2024) for negative staining. TEM images were obtained using a JEOL JEM 1400-Flash instrument (Peabody, MA, USA) at the University of Tennessee’s Advanced Microscopy and Imaging Center.

#### 2.2.3. In Vitro Testing of CQDs

Human adipose tissue-derived mesenchymal stem cell cultures were established from the tissues collected from patients undergoing panniculectomies, in accordance with a protocol approved by the Institutional Review Board at the University of Tennessee Medical Center (Protocol # 3995). Previously isolated, characterized, and cryobanked green fluorescent protein (GFP) tagged adipose tissue-derived human MSCs were used to evaluate the cytocompatibility and adherence of S1 and S2 CQDs [36]. Adult MSCs collected at passages 3–5 were used.

Glass coverslips (15 mm diameter and 0.15 mm thick) (Bellco Glass, German 1, Vineland, NJ, USA) were first coated with PEI as described above. The coverslips were cleaned with isopropyl alcohol and dried, and then were submerged in the PEI solution for 24 h. Then, 150 µL of S1 and S2 dispersions containing 1.37 × 10^−4^ g·mL^−1^ were drop-coated onto each glass coverslip using a pipette. Coated coverslips were dried for 24 h at 80 °C. Substrates were sterilized using hydrogen peroxide (Sterilis, Sterilis Solutions, Boxborough, MA, USA) for biological evaluations. 

Silicon-coated substrates were used to investigate cell attachment to the CQDs and assess cytocompatibility, with blank silicon cleaned with isopropyl alcohol used as a control. Four groups of samples were prepared prior to cell seeding. These included Si, Si + PEI, Si + PEI + S1, and Si + PEI + S2. Samples containing PEI were soaked in PEI solution, as described previously. Then, 150 µL of S1 and S2 were each drop coated onto Si + PEI samples. The samples were dried for 24 h at 80 °C. Roughly 50,000 GFP-tagged hMSCs were onto each of the four substrates. Cells were imaged after 24 h.

Next, to assess cell adherence and evaluate the overall cell shape and structure as an indication of cytocompatibility on CQD surfaces, 50,000 hMSCs in a volume of 150 μL were seeded onto PEI/nanoparticle-coated glass coverslips. These cells were not tagged with GFP. The 150 μL volume of cell suspension was sufficient to cover the coverslip completely without any overflow, thus making sure that all the cells were seeded onto the coated substrates only. Cells were allowed to attach for at least 1 h before adding the complete growth media for incubation. Cells were incubated at 37 degrees, 5% CO_2_, and were fixed after 24 h and 7 days. Subsequently, immunofluorescence using Alexafluor-labeled Phalloidin (Abcam, Cambridge, UK) was used to evaluate the cytoskeletal organization of cells using methods reported earlier [21].

## 3. Results

### 3.1. Lignin-Derived CQDs Exhibit Typical Features of Nanoparticles

**Figure 1:**
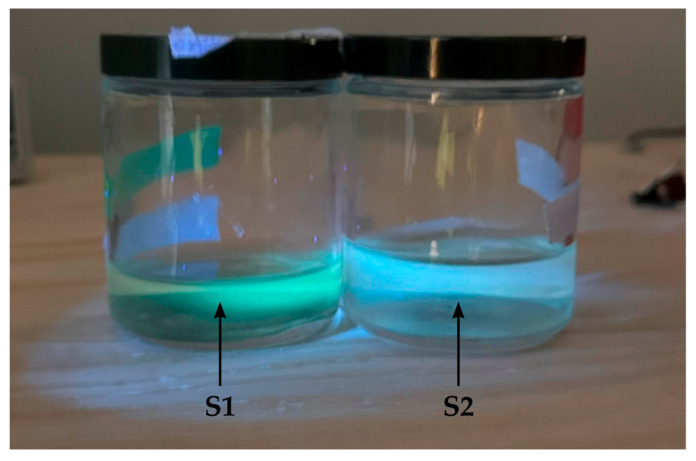
UV fluorescence of CQDs. Representative figure to show the specific fluorescence of S1 and S2 lignin-derived CQDs. CQDs were suspended in milli-q-water. Nanoparticles were imaged under a 365 nm wavelength UV light. Differences in the fluorescence confirm the differences between S1 and S2.

S1 and S2 forms of CQDs were successfully fabricated, as confirmed by the blue emission under UV light (Figure 1). Carbon nanoparticles displaying fluorescence emission during excitation through the use of the 365 nm UV light is a product of the quantum confinement effect, which is one of the core properties of quantum dots [37,38,39].

**Figure 2:**
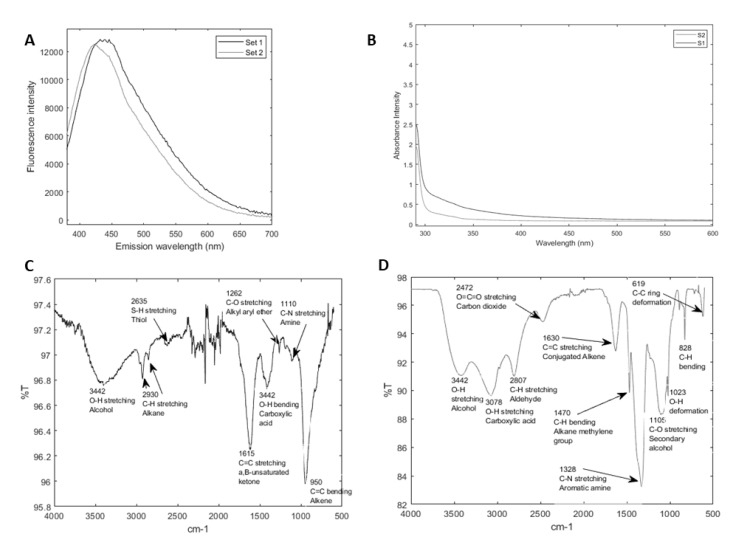
Spectral analyses of CQD nanoparticles. Representative data showing the Emission spectra of S1 and S2 with an excitation wavelength of 350 nm (**A**), the absorbance spectra of S1 and S2 (**B**), and the FTIR spectra of S1 (**C**) and S2 (**D**), Arrows in the FTIR spectra indicate the wavenumbers where significant peaks were observed, as well as the corresponding bond and the functional group.

The observed fluorescence of CQDs in Figure 1 was further confirmed by their spectral analyses. The emission and absorbance spectra of both S1 and S2 are illustrated in Figure 2A,B. The emission spectra of S1 and S2 aqueous solutions were obtained with a 350 nm excitation wavelength (Figure 2A). S1 exhibited an emission peak at 450 nm, whereas S2 displayed an emission peak at 405 nm. The blue shift of the emission peaks of S1 to S2 noticeable under the same excitation wavelength, supported the data from Figure 1. This shift was consistent and was observed with each batch of S1 and S2. Since the photoluminescence shift of nanoparticles can be attributed to variations in surface properties, molar absorptivity, and carbon dot size [15,40,41], identifying a specific factor causing this shift requires further investigation, which is beyond the scope of this study. Additionally, we also observed that S2 exhibited a slightly lower emission intensity compared to S1. The absorbance spectra of S1 and S2 aqueous solutions (Figure 2B) were similar over the range of 300–500 nm. Dispersion S2 exhibited slightly lower absorbance, which could also be due to the factors described above. Based on the fluorescence and absorption spectral analyses, we confirmed that we successfully synthesized two distinct forms of lignin-derived CQDs.

Nanoparticles possess unique physical properties. The principal parameters typically used to characterize nanoparticles are their elemental composition, shape, size, toxicity, and surface charge [42]. In this study, we first evaluated the bonded species of the S1 and S2 CQDs using FTIR spectroscopy. Specifically, FTIR spectroscopy is a versatile technique for the characterization and understanding of the surface functional groups present on the CQDs. Functional groups determine the shape of the nanoparticles, which in turn determine their functions. Figure 2C,D show FTIR spectra of S1 and S2. Labeled in the figures are wavenumbers where significant peaks were observed and the associated bond and functional group. FTIR revealed similar functional groups between S1 and S2, including alcohols, alkenes, carboxylic acid, and amine groups. Dissimilarly, S2 showed a significant peak at 1328 cm^−1^ as indicated, correlating to the stretching of C-N aromatic amines. This linkage also suggests the existence of aromatic amine groups in S2 and, thus, potentially higher nitrogen content relative to S1. In our study, this variation in the surface chemistry becomes relevant in evaluating cellular adhesion and proliferation, as described in the later section.

Next, the zeta potential of each form of CQDs was measured to evaluate their surface charge. The average zeta potential of S1 was −26.46 mV, and that of S2 was −14.7 mV, suggesting the anionic nature of the two forms. Both these values fall between −30 mV and 30 mV, suggesting that the nanoparticles had sufficient repulsive force from one another to retain colloidal stability [43]. Comparatively, the S2 CQDs exhibited a relatively lower zeta potential, suggesting that the S2 CQDs are potentially more prone to aggregate due to attractive van der Waals forces between the nanoparticles [38].

**Figure 3:**
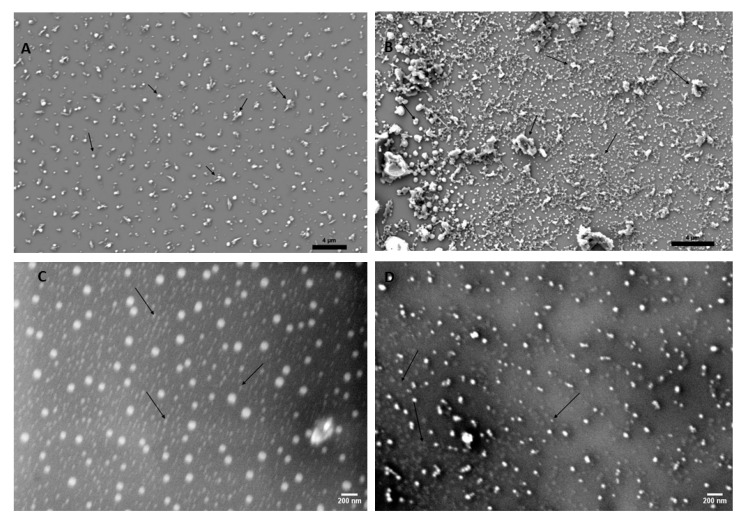

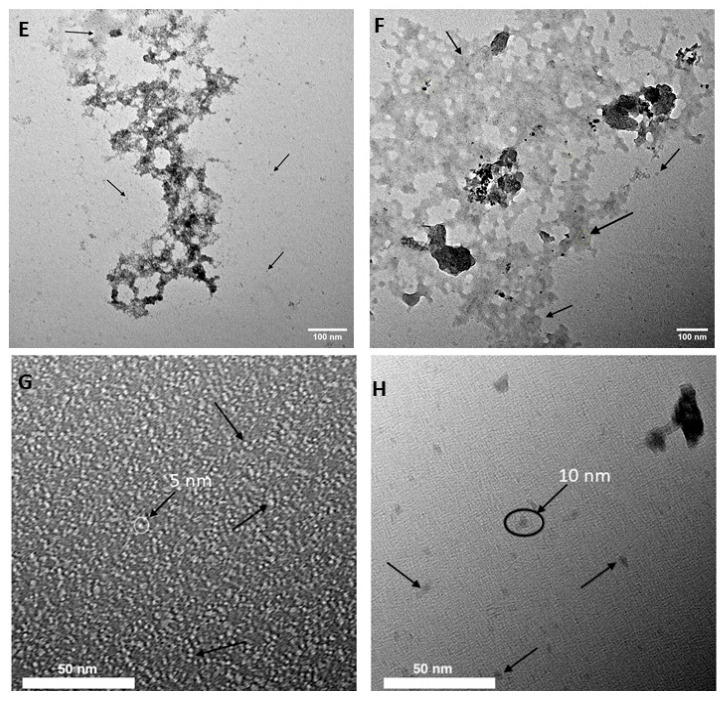
Microscopy of CQDs. Representative images showing the SEM and TEM of S1 (**A**,**C**,**E**,**G**) and S2 ‎‎(**B**,**D**,**F**,**H**), respectively. Arrows in (A,B) indicate S1 and S2 aggregates, respectively; Arrows in ‎‎(**C**,**D**) indicate individually dispersed S1 and S2 CQDs, respectively using SEM. Similarly, arrows in ‎‎(**E**,**F**) indicate S1 and S2 aggregates, respectively; Arrows in (**G**,**H**) indicate individually dispersed S1 ‎and S2 CQDs, respectively using TEM.

Finally, SEM and TEM were used to characterize the morphology, and size distribution of both S1 and S2 nanoparticles. SEM and TEM imaging showed that S1 and S2 both had a tendency to form aggregates when drop-coated onto flat substrates. Figure 3A,C show the SEM of S1, and ‎Figure 3‎B,D show the SEM of S2. The TEM of S1 is shown in ‎Figure 3‎E,G, and the TEM of S2 is shown in ‎Figure 3‎F,H. Individual CQDs were observed at higher magnification, while aggregates of the nanoparticles were observed at a lower resolution. Subjectively, aggregation was more pronounced in S2 coating. Aggregate size ranged between 100 nm to 2 μm. Individual CQD sizes of both S1 and S2 ranged from 5 nm to 10 nm, which is well within the size range of fluorescent quantum dots [38,44,45]. According to both SEM and TEM, both S1 and S2 CQDs were dispersed between the aggregates, demonstrating that the nanoparticles were distributed throughout the substrates.

### 3.2. S1 and S2 CQDs Support Cell Attachment

**Figure 4:**
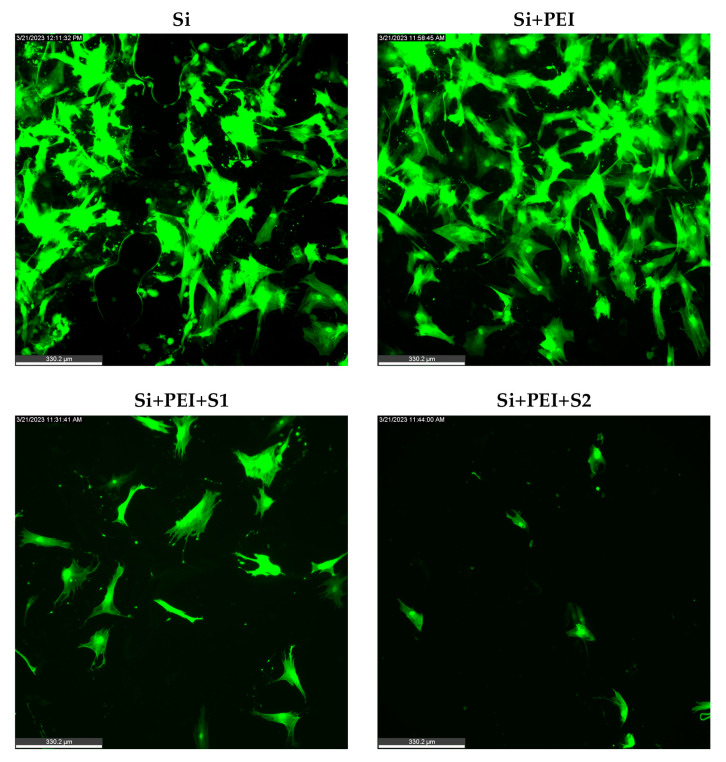
Cell adherence on CQDs. Representative images showing the attachment of GFP-tagged hMSCs on Si, Si + PEI, Si + PEI + S1, Si + PEI + S2 substrates. Note the relatively few numbers of cells on the Si and Si + PEI coated substrates relative to the substrates coated with S1 and S2 CQDs. Scale bar = 330.2 µm.

Previously generated and characterized primary cultures of human MSCs tagged with the GFP protein were used to evaluate cellular attachment to both S1 and S2 CQDs (Figure 4). Fluorescently tagged cells were used to confirm that the cells attach to the substrates coated with S1 and S2 CQDs only and not to silicon (Si) alone or Si + polyethyleneimine (PEI). Cell attachment to silicon substrates containing S1 and S2 CQDs was observed as early as 24 h.

### 3.3. S1 and S2 CQDs Are Cytocompatible

**Figure 5:**
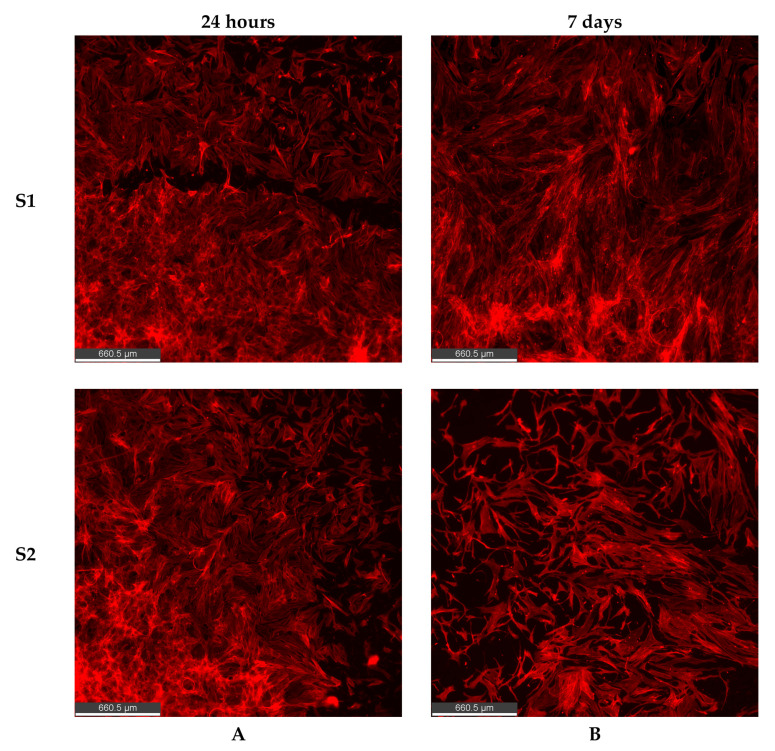
F actin expression of hMSCs on S1 and S2 CQD substrates. Representative images to show cell adherence on S1 and S2 CQDs after 24 h (**A**) and 7 days (**B**). Images show healthy cell morphology and structure at both time points. Subjectively there is a difference in cell morphology and patterns in which the cells cluster with time. This is a future line of investigation that is beyond the scope of this study. Scale bar = 660.5 µm.

Phalloidin staining of filamentous actin expressed by a healthy and viable cell shows the overall cell shape and structure. Cell shape is an indicator of cell health [46]. Any change in the normal cell shape predicts a change in the cell, which could potentially lead to changes in function, or merely suggest that cells are not healthy. Cells undergo morphological changes relating to differences in the spatial distribution and expression of key cytoskeletal proteins, F actin being the major one [47,48]. Phalloidin is a highly selective bicyclic peptide that binds to all variants of actin filaments and helps us understand the cytoskeletal organization of cells onto substrates. As a result, we next evaluated the expression of F-actin using phalloidin staining when MSCs adhere to S1 and S2 CQDs. Cells were evaluated at 24 h and then at 7 days after seeding. F actin expression at 24 h proved cell attachment, which was supported through day 7, confirming the cytocompatibility of S1 and S2 CQD coated substrates (Figure 5). Cells displayed elongated bodies with distinct cell-to-cell communication.

## 4. Discussion

In this study, we present data to show that we successfully generated two forms of lignin-derived CQDs using a bottom-up approach. Blue fluorescence of both S1 and S2 CQDs was observed when exposed to 365 nm UV light, which confirmed the synthesis of nanoparticles. This was used to validate every batch of newly synthesized nanoparticles before use.

We did observe several similarities and dissimilarities in their material characteristics. Most striking were the differences in photoluminescent properties between S1 and S2 may be due to variances in molar absorptivity which will be further investigated through spectrophotometry analysis. Photoluminescence together with the particle size measured by TEM further confirmed that both S1 and S2 CQDs are nanoparticles and hence, can be referred to as carbon quantum dots [38,39].

FTIR plots of both S1 and S2 showed the commonly observed functional groups such as alcohols, alkanes, and alkenes. Notably, only the S2 CQDs contained aldehyde and aromatic amine groups. Furthermore, the S2 CQDs showed lower zeta potential, suggesting a relatively higher tendency for aggregation. This was supported by SEM. Images showed aggregation of both S1 and S2, with S2 displaying slightly larger aggregates. S1′s largest aggregates were observed at a scale of 0.5–1 μm. S2′s largest aggregates were observed at a scale of 1–2 μm. TEM imaging showed both CQDs to range between 5–10 nm.

Nanoparticle aggregation is supported by the theory of “blackened carbon” proposed by López-de-Uralde et al. [49]. It is believed that commercially available pyrolyzed carbon clusters in solution form aggregates and agglomerations ranging from 50 nm to over 1 µm. Bearing this in mind, the S2 form of CQDs does display slightly larger aggregates compared to S1 CQDs, likely attributed to the difference in zeta potential. It is believed that the novel technique of soaking pyrolyzed lignin in nitric acid at 80 °C for 8 h induces nitrogen content into S2 and introduces amine groups on the S2 CQDs, which may lead to changes in the material properties and the biological response. Based on the performed material characterization, it is confirmed that there is a difference between S1 and S2 in terms of emission spectra and present functional groups most likely due to nitic acid treatment. (Figure 1, Figure 2, Figure 3 and Figure 4).

When hMSCs were seeded onto various substrates, as described in Figure 4, silicon and silicon + PEI substrates showed a significantly reduced cell presence after 24 h compared to substrates drop-coated with S1 or S2 CQDs. This suggests a lack of cytotoxicity of the nanoparticles and the PEI. This also suggests that the CQD coating potentially promotes cell attachment. The observed adherence and viability of GFP-tagged hMSCs on substrates coated with S1 and S2 CQDS can therefore be attributed to the presence of CQDs. These observations are supported by published reports from other laboratories [29,50]. Mezhevikina et al. showed that the presence of PEI as a substrate coating does not impact cell adhesion and proliferation. Praseetha et al. reported that beetroot extract-derived CQDs of negative surface charge have good affinity for a cell and hence, serve as acceptable substrates for cell–particle binding. Taken together, published literature and data presented in this study show that CQDs as coatings are cytocompatible.

Surface treatments with nitric acid are a relatively quick and easy method used to produce carbon-based nanomaterials that are cytocompatible and contain nitro-functional groups on the surface [51]. This is because the presence of amino groups on the surface of nanomaterials promotes cell adherence, and proliferation and affects differentiation. Specifically, the positive charge on the amino groups can attract the negatively charged biomolecules such as proteins or DNA in a cell in aqueous media under physiological conditions [52]. Hence, we decided to use this relatively simple method to produce two different forms of CQDs. Interestingly, both the forms were cytocompatible and the material characterization did identify variations due to nitrogen doping. Further experiments to evaluate any effect on cell response will be performed.

Using Phalloidin-labelled F-actin expression of hMSCs, healthy cytoskeletal organization on both the S1 and S2 substrates was observed, confirming cytocompatibility (Figure 5). Cytoskeleton is a highly dynamic network of proteins including actin and tubulin and the spatial arrangement of these proteins is dependent on the cell type and state [53]. Cytoskeletal rearrangement, which might include a change in the overall shape and structure, indicates a change in the microenvironment of the cell referred to as mechanotransduction or mechanosignalling and in tissue engineering, typically occurs in response to the properties of the biomaterials used [54,55]. We have previously reported that there are distinct changes in F-actin expression when hMSCs adhere and undergo differentiation on graphene surfaces relative to a tissue culture polystyrene surface [21]. MSCs growing on a tissue culture polystyrene surface maintain their fibroblastic shape and hence, any change in their shape, due to an external stimulus, suggests differentiation to specific lineages [56,57]. These changes can be subjectively evaluated using F-actin staining. Future studies aim to identify these changes, if any in differentiation pathways and cell lineages. Additionally, at the in vitro level, altering the concentration and particle distribution of S1 and S2 CQDs and evaluating the impact on hMSC behavior will be conducted. This platform provides us with an opportunity to evaluate the response of not only MSCs but any cell type to CQDs, in vitro prior to in vivo applications. This study also presents a foundation for understanding the role(s) of lignin-derived CQDs in regenerative medicine and tissue engineering.

Further studies to evaluate stem cell response to lignin-derived CQDs are being conducted. This includes altering the distribution of CQDs coated on in vitro surfaces to create various CQD dispersions with differences in concentration, coating thickness, topography, and uniformity. Multiple coating and blending techniques to incorporate lignin-derived CQDs into tissue engineering scaffolds for future in vivo implantation are also being investigated. Further characterization of material properties of lignin-derived CQDs will further our understanding of S1 and S2. This will be carried out using atomic force microscopy (AFM), nanoscale infrared spectroscopy (nano-IR), electron energy loss spectroscopy (EELS), further S/TEM analysis, energy dispersive spectroscopy (EDS) and X-ray diffraction (XRD) will allow further insight into CQD composition, structure, and topography of CQD coatings/dispersions. Correlating these material properties with cell response to CQD coatings will further the understanding of the mechanisms of mechano-signaling, and thus function. These lines of current and future investigations will support the role of lignin-derived CQDs in biomedicine.

## Data Availability

All raw data are available on request.
